# Supplementary material for: Securing digital images: A chaos-driven scrambling algorithm using the Rössler system
Source: PLoS One. 2025 Jun 25;20(6):e0326202. doi: 10.1371/journal.pone.0326202 (PMC12193603; doi:10.1371/journal.pone.0326202)
Supplement: Attachment — Submitted filename: PONE-D-25-11205.pdf [file pone.0326202.s001.pdf]

The manuscript titled **"Reinforcing Image Security: A High-Entropy Scrambling Algorithm Using the Rössler Chaotic System"** lacks significant contribution to the field of image encryption and security. The work does not demonstrate sufficient novelty, as similar approaches using chaotic systems for image encryption have been extensively explored in the literature. Additionally, the manuscript requires substantial improvement in its writing and focus. For instance, in **Section 3, "Proposed Image Encryption,"** the authors spend excessive time explaining general cryptographic concepts rather than focusing on the core methodology and explaining unnecessary points of their proposed algorithm. Below are some suggestions that can be considered to improve the overall quality of the research.

1. The manuscript's title, "*Reinforcing Image Security: A High-Entropy Scrambling Algorithm Using the Rössler Chaotic System*," emphasizes high entropy as a key feature. However, the reported entropy value of 7.998 for the encrypted images does not demonstrate a significant improvement over existing schemes, some of which achieve entropy values as high as 7.9999.
2. In the abstract, the symbol "&" is not valid; instead, write "and."
3. What are the major contributions or innovative points? I suggest writing a major contribution to this research at the end of the introduction section.
4. R¨ossler Chaotic System has already been developed and is being used in image encryption schemes, so why are you discussing it unnecessarily instead of utilizing it for your methodology and citing the particular research?
5. Section 3 and subsection 3.2 are the same!
6. In section 3.1, a new stream of random numbers $\{\text{ hori }_t\}_{t=1}^{mn}$, $\{\text{ verti }_t\}_{t=1}^{mn}$ and $\{\text{ mask }_t\}_{t=1}^{mn}$, are generated through raw streams. The generated numbers should undergo statistical tests (NIST Statistical Test Suite, Run Test, Frequency Test, Autocorrelation Test or Entropy etc.) to validate their randomness. I recommend incorporating one or more of the following tests to ensure the robustness of the random number streams.
7. In section 3.2, What I got the authors have utilized the Rossler Chaotic System for creating streams of random numbers. These random numbers are then tailored (using Equation 2)

to fit the algorithmic logic of pixel scrambling. Later, the described process of row-based scrambling of the image, and then $\boldsymbol{cipher = img_2\ (i) \oplus mask\ (i)}$! Is it not very simple encryption? How will it resist statistical attacks or known and chosen plaintext attacks and post-quantum attacks?

8. Fig. 2 is unnecessarily stretched to a full page and could be more effectively resized to fit half a page.

9. Since the pseudocode/algorithm is already provided in the manuscript, it's unnecessary to explain every single step of that again in detail in Section 3.2.

10. Ensure the values in Table 1 are accurate and consistent with the sources used for comparison. Additionally, since the secret key size is a crucial factor in encryption algorithms, does the relatively smaller key size of the proposed algorithm undermine its security strength?

11. Encryption schemes are evolving rapidly. Why are the authors comparing results with older schemes from 2012 and 2018 instead of more recent ones from 2022 onwards?

12. In Table 12, what is meant by *O-C* and *O-D*?

13. Table 13 shows that the proposed method takes an average of 1.22 seconds to encrypt a 256×256 grayscale image. In today's digital era, where image sizes and formats (such as RGB instead of grayscale) have significantly expanded, and numerous recent robust methods can encrypt RGB images in microseconds, this raises concerns about the usability and validity of the proposed algorithm. Does the slower processing time diminish its practicality and relevance in comparison to faster, more advanced alternatives?
