# Supplementary material for: Securing digital images: A chaos-driven scrambling algorithm using the Rössler system
Source: PLoS One. 2025 Jun 25;20(6):e0326202. doi: 10.1371/journal.pone.0326202 (PMC12193603; doi:10.1371/journal.pone.0326202)
Supplement: Attachment — Submitted filename: Answer to Reviews.docx [file pone.0326202.s002.docx]

**Reviewer #1:**

1) Manuscript employs the 3D Rossler chaotic system for random number generation, but it does not mathematically justify why this system is preferable over other chaotic maps such as Logistic, Henon, or Chen systems. A Lyapunov exponent analysis, bifurcation diagram, and entropy measure comparison should be added to establish its superiority in key sensitivity and randomness properties.

Ans: Concern has been addressed. Kindly see the Figures 5 and 6.

2) Proposed scrambling approach relies on row- and column-based pixel transpositions but lacks a formal cryptographic security proof. The authors should mathematically demonstrate the diffusion and confusion properties using metrics like Shannon entropy, differential uniformity, and avalanche effect.

Ans: Kindly see the Section 6.9 for Shannon entropy and 6.11 for avalanche effect.

We appreciate the reviewer’s suggestion regarding differential uniformity analysis. In our study, we have already assessed the impact of minor changes in the plaintext on the ciphertext using established metrics such as the Avalanche Effect, NPCR (Number of Pixels Change Rate), UACI (Unified Average Changing Intensity) and key sensitivity analysis. These measures effectively capture the core idea of differential uniformity in the context of image encryption by quantifying the algorithm’s sensitivity to small input variations.

3) Proposed claim that the encryption scheme offers a large key space is not substantiated with a thorough key sensitivity analysis. A detailed parameter space exploration (i.e., how changes in initial conditions of the chaotic system affect encryption security) and a comprehensive key space growth analysis are required.

Ans: Kindly see the new section 6.2 regarding the key sensitivity.

4) The paper focuses on encryption speed as a usability metric but fails to analyze computational efficiency, energy consumption, and real-time feasibility on IoT devices. Include it in the revised manuscript.

Ans: For computational efficiency, kindly see Table 19. Moreover, time complexity O(8n^2^) has also been calculated. For energy consumption, kindly see the Table 6. Kindly see the conclusion section for “real-time feasibility on IoT devices.”

5) The manuscript does not sufficiently compare the proposed encryption technique against state-of-the-art where high entropy based ciphers images like
DNA based work - Enhancing Image Security via Block Cyclic Construction and DNA Based LFSR
Moore automation- An Applied Image Cryptosystem on Moore’s Automaton Operating on δ (qk)/ 2
LFSR based work- Chaos-based medical image encryption scheme using special nonlinear filtering function based LFSR

Ans: Concern has been addressed.

6) The proposed scheme is tested on standard test images like Lena and Baboon, but real-world image datasets (e.g., medical, satellite, and biometric images) should be included.

Ans: According to the journal's policy, the inclusion of copyrighted images without prior permission is not permitted. Therefore, we respectfully apologize to the esteemed reviewers for this oversight.

**Reviewer #2:**

About the manuscript “Reinforcing Image Security: A High-Entropy Scrambling Algorithm Using the Rossler Chaotic System”, I think this theme is a hot academic topic. Overall, it sounds technically feasible, and the structure of the manuscript is complete. Yet, here are some suggestions which should be taken into account to improve the current version.

1.Abstract and conclusion should be rewritten as they do not effectively summarize the entire paper. For instance, in the abstract, most of the descriptive language is about the background, which confuses the reader. It should primarily introduce the author's technical solution, key points of the method, and the superior characteristics of the proposed method.

Ans: Abstract has been revised to address the respected reviewer’s concern.

2.The title is about the High-Entropy Scrambling Algorithm, but how is High-Entropy reflected? What testing methods are used to verify, or how is it compared with existing algorithms? As far as I know, Scrambling is just a position-based shuffling.

Ans: Given the respected reviewer’s comment, we have changed the title. The new title is Securing Digital Images: A Chaos-Driven Scrambling Algorithm Using the R\"{o}ssler System. We have used the following testing methods to authenticate the novel algorithm.

i. Key space
ii. Key sensitivity
iii. Statistical Randomness Analysis
iv. Irregular deviation and maximum deviation security analyses
v. Energy and contrast analyses
vi. Homogeneity analysis
vii. Statistical analysis (Cartesian histogram analysis, Polar histogram analysis, Correlation analysis)
viii. Information entropy
ix. Local Shannon entropy (LSE)
x. Anti-jamming Capability Analysis
xi. Avalanche (Differential) Attack Analysis
xii. Ciphertext-only, Known plaintext, Chosen-plaintext and Chosen-ciphertext attacks Analysis
xiii. PSNR and SSIM analyses
xiv. Speed and time complexity analyses

3.The font size of the coordinate axes in Figure 1 is too small, making it difficult to read. In fact, all figures in the paper have similar issues. It is recommended to uniformly modify them, improve the resolution and quality of the figures, making them highly readable.

Ans: Concern has been addressed.

4.Figure 2 seems to be the algorithm flowchart of the entire paper? But just from this figure, it is confusing and unclear. From the context of the paper, it should be an image encryption algorithm, and shuffling is the main step. It is suggested that all the main encryption steps should be expressed and uniformly drawn in Figure 2. In other words, Figure 2 is the framework diagram of the entire algorithm.

Ans: Since row-based and column-based scramblings are symmetric in their character and orientation, so drawing only will be sufficient. The other can be understood analogously. Kindly see the manuscript at the very start of the Section 4.2, “The image scrambler consists of two algorithms, i.e., Row based scrambling and Column based scrambling. Since, Row-Based and Column-Based scramblings are symmetric, so we have drawn only the schematic diagram of how the row based scrambling has been carried out.”

5.Regarding Figure 8, it looks quite novel and interesting. But I am not clear about how this figure is generated, are there any relevant references or websites? Or executable code? It is hoped that the details can be provided as much as possible so that peers have the opportunity to reproduce the related results.

Ans: Kindly see the work “[Utilizing the nth root of numbers for novel random data calculus and its applications in network security and image encryption](https://www.sciencedirect.com/science/article/pii/S0957417424028598)” for details. Moreover, below is the Python code.

import numpy as np

import matplotlib.pyplot as plt

from skimage import io

\# Load the plain and cipher images of Lena

plain_image = io.imread('chair.png')

cipher_image = io.imread('chair_out.png')

\# Function to compute and draw histograms in polar coordinates

def plot_polar_histogram(image, title, ax):

\# Split the image into R, G, B channels

r, g, b = image[:, :, 0], image[:, :, 1], image[:, :, 2]

\# Compute histograms

hist_r, bins_r = np.histogram(r.flatten(), bins=256, range=[0,256])

hist_g, bins_g = np.histogram(g.flatten(), bins=256, range=[0,256])

hist_b, bins_b = np.histogram(b.flatten(), bins=256, range=[0,256])

\# Normalize histograms

hist_r = hist_r / np.sum(hist_r)

hist_g = hist_g / np.sum(hist_g)

hist_b = hist_b / np.sum(hist_b)

\# Plot in polar coordinates

theta = np.linspace(0, 2*np.pi, 256)

ax.plot(theta, hist_r, color='red', linewidth=1.5, label='Red')

ax.plot(theta, hist_g, color='green', linewidth=1.5, label='Green')

ax.plot(theta, hist_b, color='blue', linewidth=1.5, label='Blue')

ax.set_title(title, fontsize=12)

ax.set_theta_zero_location('N')

ax.set_theta_direction(-1)

ax.legend(loc='upper right')

\# Create a figure for the plots

fig, axs = plt.subplots(1, 2, subplot_kw={'projection': 'polar'}, figsize=(14, 7))

\# Plot histograms for the plain and cipher images

plot_polar_histogram(plain_image, 'Polar Histogram of Plain Image', axs[0])

plot_polar_histogram(cipher_image, 'Polar Histogram of Cipher Image', axs[1])

plt.show()

6.Regarding the discussion on cryptanalysis in Section 5.7, it is recommended to refer to some systematic cryptanalysis work, such as:
<https://doi.org/10.1016/j.eswa.2024.123748>. <https://doi.org/10.1016/j.eswa.2023.121514>.
From the above cryptanalysis work, it is seen that the current algorithm is difficult to resist chosen plaintext attacks.

Ans: Concern has been addressed.

7.Moreover, experimental analysis and results discussion are relatively little, and the enrichment is recommended. At the same time, it is suggested that some of the latest and similar work should be discussed in the introduction, such as in the security enhancement design, which has similarities and enlightening value.
DOI :10.3390/math12243917; DOI:10.1016/j.eswa.2024.123190;
DOI: 10.1007/s11071-021-06206-8; 10.1007/s00371-023-02812-2。

Ans: Concern has been addressed.

8.The current article draft still contains a significant number of typographical and grammatical errors. Additionally, it is recommended to study the writing and expression of higher-level literature, and to carefully check and correct these issues.

Ans: Concern has been addressed.

In summary, the current draft is unacceptable and cannot be published, thus my review opinion is major revision. Consider the next step after the revisions are made.

**Reviewer #3:**

Work is nice. A new technique based on the rows and columns in the scrambled image, has been developed. But I have few concerns.

1. Manuscript is furnished with language and grammar issues which must be addressed.

Ans: Concern has been addressed.

2. The advantages that this technique brings, must be described in a very clear way.

Ans: Kindly see the tale of the Introduction section where contributions of this study have been described in a bullet form.

3. Homogeneity analysis needs to be added in the security analysis section.

Ans: Concern has been addressed. Kindly see the Section 6.6.

4. NIST random number analysis should be included.

Ans: Concern has been addressed (Section 6.3).

5. Irregular deviation analysis should be added in the analysis section.

Ans: Concern has been addressed (Section 6.4).

6. Introduction section is weak. It is not developing context sufficiently. The related work should be added.

Ans: A new section of Related work has been added.

**Reviewer #4:**

This manuscript introduces a new image scrambling algorithm that employs row- and column-based approaches. It involves inserting input image columns into a 2D scrambled image, repeating the process for rows, and enhancing diffusion through an XoR operation with random number streams generated by the 3D Rössler chaotic system. Both simulation and security analysis demonstrate the cipher's resilience to various cryptanalytic attacks, recommending it for real-world applications.

Overall, the presented work exhibits a certain degree of novelty and contribution. However, the manuscript has noticeable deficiencies in several aspects. I strongly recommend that the authors carefully revise and improve the manuscript based on the review comments.

1). The abstract does not meet the general requirements. Please refer to A novel multi-channel image encryption algorithm leveraging pixel reorganization and hyperchaotic maps and Exploiting robust quadratic polynomial hyperchaotic map and pixel fusion strategy for efficient image encryption for guidance on enhancements. The authors should concisely elaborate on the research background in the abstract, identify the research gap, outline the main content of their work (including key steps), present the key findings, and emphasize the superiority and significance of their contributions.

2). It is recommended that the authors make appropriate adjustments to the list of keywords. Typically, we should meticulously choose five to seven keywords that accurately capture the core content and distinctive attributes of the manuscript, while maintaining simplicity to optimize retrievability. Please refer to Exploiting newly designed fractional-order 3D Lorenz chaotic system and 2D discrete polynomial hyper-chaotic map for high-performance multi-image encryption and A robust memristor-enhanced polynomial hyper-chaotic map and its multi-channel image encryption application for further guidance. For instance, in my view, "Image encryption," "Pixel scrambling," and "Secure analysis" should at least be selected as keywords.

3). The current introduction is overly concise and lacks necessary content. The authors should enumerate various emerging image encryption algorithms or schemes, such as those based on DNA computing, quantum computing, optical transformations, chaotic systems, neural networks, compressive sensing, and so forth, and introduce some of the latest advancements in the field of image encryption. They should identify research gaps and discuss the motivations and advantages of choosing specific techniques and methods for image encryption. Some noteworthy related works include Dynamic analysis and implementation of FPGA for a new 4D fractional-order memristive hopfield neural network, An image encryption algorithm based on Tabu search and hyperchaos, A novel multi-channel image encryption algorithm leveraging pixel reorganization and hyperchaotic maps, Exploiting robust quadratic polynomial hyperchaotic map and pixel fusion strategy for efficient image encryption, and Exploiting newly designed fractional-order 3D Lorenz chaotic system and 2D discrete polynomial hyper-chaotic map for high-performance multi-image encryption.

4). Cryptanalysis plays a pivotal role in ensuring the rationality, practicality, and security of image encryption algorithms or schemes. Therefore, it is recommended that the authors dedicate a separate paragraph in the introduction to introduce and discuss some recent advancements in cryptanalysis research related to image encryption, such as Cryptanalysis of an image encryption algorithm using quantum chaotic map and DNA coding, Cryptanalyzing an image cipher using multiple chaos and DNA operations, Cryptanalysis and improvement of the image encryption scheme based on Feistel network and dynamic DNA encoding, and Cryptanalysis and improvement of the image encryption scheme based on 2D Logistic-Adjusted-Sine map. Furthermore, a rigorous validation of the proposed image encryption scheme's rationality, practicality, and security should be conducted in light of these cryptanalysis studies.

5). To strengthen the clarity and impact of the manuscript, I suggest that the author concisely highlight the significance and contributions of their work at the end of the introduction, utilizing a bullet-point list format. Please refer to A novel multi-channel image encryption algorithm leveraging pixel reorganization and hyperchaotic maps and Exploiting robust quadratic polynomial hyperchaotic map and pixel fusion strategy for efficient image encryption for guidance on revisions.

6). The manuscript necessitates refinement in the presentation and elucidation of the equations and mathematical symbols. For improvements, please kindly refer to Exploiting newly designed fractional-order 3D Lorenz chaotic system and 2D discrete polynomial hyper-chaotic map for high-performance multi-image encryption and A secure and efficient image transmission scheme based on two chaotic maps.

7). In Section 3, the organization of the content appears somewhat disorganized, lacking necessary details and adequate descriptions. Please refer to A novel multi-channel image encryption algorithm leveraging pixel reorganization and hyperchaotic maps and Exploiting robust quadratic polynomial hyperchaotic map and pixel fusion strategy for efficient image encryption for guidance on improvements. Furthermore, for each encryption step, the authors are requested to clearly articulate the design background, rationale, motivation, process, or decisions underlying it. Otherwise, it may give the impression that the authors are merely arbitrarily stacking encryption steps without due consideration for the rationality, practicality, security, and efficiency of the design.

Ans: For all the above concerns, the relevant works have been cited.

8). Many figures in the manuscript, such as Figure 2, are of extremely poor quality, containing evident errors and deficiencies. Clearly, they do not meet the quality standards expected of a rigorous scientific research paper. It is recommended that the authors refer to relevant high-quality papers for improvements.

Ans: Concern has been addressed.

9). In the experimental section, the authors should clearly specify the hardware and software configurations, as well as the benchmark datasets used for their experiments. Please refer to A secure and efficient image transmission scheme based on two chaotic maps and Exploiting robust quadratic polynomial hyperchaotic map and pixel fusion strategy for efficient image encryption for improvements. This will help ensure the evaluability and reproducibility of their experimental results.

10). In Section 4, the authors have not conducted a systematic and comprehensive security evaluation and efficiency analysis of the proposed encryption scheme. Please refer to relevant high-quality papers to improve the details, data, analysis, and discussion for each evaluation item. Furthermore, the authors should compare their proposed encryption scheme with some of the latest encryption schemes to highlight its advantages.

Ans: Relevant researches have been cited.

11). While considering security, encryption efficiency is also an important factor that researchers must consider. Therefore, it is recommended that the authors analyze and discuss the encryption efficiency of the proposed image encryption algorithm. Please refer to A novel multi-channel image encryption algorithm leveraging pixel reorganization and hyperchaotic maps and A secure and efficient image transmission scheme based on two chaotic maps. Besides, it is recommended to conduct extensive experiments using test images of different sizes (such as 256×256, 512×512, and 1024×1024) to enrich the experimental data. In addition, the obtained experimental data should be compared with some state-of-the-art encryption algorithms or schemes.

Ans: Concern has been addressed. Kindly see the Figure 10.

12). The conclusion section does not meet the requirements. Please kindly refer to A novel multi-channel image encryption algorithm leveraging pixel reorganization and hyperchaotic maps and Exploiting robust quadratic polynomial hyperchaotic map and pixel fusion strategy for efficient image encryption for improvements. The authors should present current problems, solutions, contributions, findings, limitations, and prospects for future work in a more engaging manner.

13). The authors are strongly advised to adjust their reference list. They should remove outdated references as much as possible and cite, introduce, analyze, discuss, and compare the latest related works, especially works related to chaotic cryptography. Outdated or low-quality references should be removed. This will help to highlight the importance, significance, superiority, and timeliness of their work.

Ans: Concern has been addressed.

14). The linguistic quality of the manuscript is suboptimal. The authors are recommended to meticulously proofread the manuscript to enhance the logicality, rigor, and consistency of the content.

Ans: Concern has been addressed.

**Reviewer #5:**

The manuscript titled **“Reinforcing Image Security: A High-Entropy Scrambling Algorithm Using the Rössler Chaotic System”** lacks significant contribution to the field of image encryption and security. The work does not demonstrate sufficient novelty, as similar approaches using chaotic systems for image encryption have been extensively explored in the literature. Additionally, the manuscript requires substantial improvement in its writing and focus. For instance, in **Section 3, “Proposed Image Encryption,”** the authors spend excessive time explaining general cryptographic concepts rather than focusing on the core methodology and explaining unnecessary points of their proposed algorithm. Below are some suggestions that can be considered to improve the overall quality of the research.

1. The manuscript’s title, “*Reinforcing Image Security: A High-Entropy Scrambling Algorithm Using the Rössler Chaotic System*,” emphasizes high entropy as a key feature. However, the reported entropy value of 7.998 for the encrypted images does not demonstrate a significant improvement over existing schemes, some of which achieve entropy values as high as 7.9999.

Ans: Thanks for your suggestion. Now, we have come up with a new title as “Securing Digital Images: A Chaos-Driven Scrambling Algorithm Using the R\"{o}ssler System”

2. In the abstract, the symbol “&” is not valid; instead, write “and.”

Ans: Concern has been addressed.

3. What are the major contributions or innovative points? I suggest writing a major contribution to this research at the end of the introduction section.

Ans: Concern has been addressed.

4. R¨ossler Chaotic System has already been developed and is being used in image encryption schemes, so why are you discussing it unnecessarily instead of utilizing it for your methodology and citing the particular research?

Ans: Many reviewers demand the justification for using the chaotic system being used. This is the reason that we have dedicated a section for this purpose.

5. Section 3 and subsection 3.2 are the same!

Ans: Thanks for your suggestion. Concern has been addressed.

6. In section 3.1, a new stream of random numbers { hori 𝑡}𝑡=1𝑚𝑛,{ verti 𝑡}𝑡=1𝑚𝑛 and { mask 𝑡}𝑡=1𝑚𝑛, are generated through raw streams. The generated numbers should undergo statistical tests (NIST Statistical Test Suite, Run Test, Frequency Test, Autocorrelation Test or Entropy etc. ) to validate their randomness. I recommend incorporating one or more of the following tests to ensure the robustness of the random number streams.

Ans: Concern has been addressed. Kindly see the new subsection 6.3.

7. In section 3.2, What I got the authors have utilized the Rossler Chaotic System for creating streams of random numbers. These random numbers are then tailored (using Equation 2) to fit the algorithmic logic of pixel scrambling. Later, the described process of row-based scrambling of the image, and then ***cipher = img2 (i)*** ⊕ ***mask (i)***! Is it not very simple encryption? How will it resist statistical attacks or known and chosen plaintext attacks and post-quantum attacks?

Ans: Although, the proposed algorithm seems very simple but paradoxically, the security analysis rendered very promising results including the known and chosen plaintext attacks. However, we do not claim that the suggested algorithm can defy the potential post-quantum attacks. We think that for this purpose, new scheme of things are required.

8. Fig. 2 is unnecessarily stretched to a full page and could be more effectively resized to fit half a page.

Ans: Concern has been addressed.

9. Since the pseudocode/algorithm is already provided in the manuscript, it’s unnecessary to explain every single step of that again in detail in Section 3.2.

Ans: Agreed but most of the readers demand a detailed explanation of the algorithms. Moreover, this is according to the state of the art.

10. Ensure the values in Table 1 are accurate and consistent with the sources used for comparison. Additionally, since the secret key size is a crucial factor in encryption algorithms, does the relatively smaller key size of the proposed algorithm undermine its security strength?

Ans: The key space of the proposed algorithms is 2^298^ which is far greater than the minimum threshold recommended by the cryptographers, i.e., 2^100^. So, we think that the proposed algorithm can withstand the potential brute force attack.

11. Encryption schemes are evolving rapidly. Why are the authors comparing results with older schemes from 2012 and 2018 instead of more recent ones from 2022 onwards?

Ans: Concern has been addressed by replacing the current works with the latest ones for the sake of comparison.

12. In Table 12, what is meant by *O-C* and *O-D*?

Ans: `O-C' represents the original and cipher images, and `O-D' denotes the original and decrypted images. This has been written in the caption of the reported table.

13. Table 13 shows that the proposed method takes an average of 1.22 seconds to encrypt a *256×256* grayscale image. In today’s digital era, where image sizes and formats (such as RGB instead of grayscale) have significantly expanded, and numerous recent robust methods can encrypt RGB images in microseconds, this raises concerns about the usability and validity of the proposed algorithm. Does the slower processing time diminish its practicality and relevance in comparison to faster, more advanced alternatives?

Ans: The observed limitation is primarily due to the use of outdated hardware resources during experimentation.
